# Effects of Nonsteroidal Anti-Inflammatory Drugs as Patient Controlled Analgesia on Early Bowel Function Recovery after Radical Cystectomy

**DOI:** 10.1038/s41598-018-22677-z

**Published:** 2018-03-15

**Authors:** Young Dong Yu, Jin Ho Hwang, Young Eun Seo, Byung Do Song, Yeon Soo Jung, Dong Hwan Lee, Sung Kyu Hong, Seok-Soo Byun, Sang Eun Lee, Jong Jin Oh

**Affiliations:** 1 Department of Urology, Seoul National University Bundang Hospital, Seongnam, Korea; 2 Department of Urology, Seoul National University College of Medicine, Seoul, Korea

## Abstract

This study aimed to evaluate the effects of ketorolac, a commonly used non-steroidal anti-inflammatory drug (NSAID) as patient controlled intravenous infusion analgesia (PCIA) for the patients underwent radical cystectomy (RC) due to bladder cancer regarding post-operational indices of recovery. Total seventy patients who underwent radical cystectomy for the treatment of bladder cancer were included in the study. 35 patients received ketorolac as PCIA (NSAIDS group) and 35 patients had morphine infusion as PCIA (morphine group). Pain intensity, bowel function recovery and length of hospital stay were evaluated. Early postoperative complications were analyzed according to surgical types (robot RC vs. open RC). Demographics were similar between two groups. NSAIDS group showed a significant reduction in postoperative vomiting (p = 0.001), time to flatus (p = 0.028), time to first bowel movement (p = 0.001) and time to first clear liquid diet (p = 0.002) compared with morphine group. No statistically significant differences were observed between two groups regarding length of hospitalization, and postoperative complications. For 48 hours after RC, pain relief was slightly better in morphine group (p < 0.001). Both open RC and robot RC cases showed significantly better bowel function recovery with NSAIDS groups. Ketorolac as PCIA is relatively effective in pain management with better gastrointestinal recovery after RC.

## Introduction

Despite improvements in surgical and aesthetical techniques, postoperative pain and ileus are still important factors that significantly influence postoperative morbidity and prolonged hospital stay that would eventually increase overall cost burden to the patients^1–3^. For high grade, non-muscle invasive muscle invasive balder cancer (NMIBC) or muscle invasive bladder cancer (MIBC), radical cystectomy (RC) with pelvic lymph node dissection is the gold standard treatment with overall survival rates of 45%^4–6^. Paralytic ileus (PI) is one of the most common postoperative complications after RC^7,8^, and PI is generally observed within 3 to 5 days post major abdominal surgery while the incidence rate is traditionally between 10–30%, even though the rates varies among different researches^9,10^. PI is associated with clinical symptoms such as nausea, vomiting, and abdominal distension and functional recovery of gastrointestinal (GI) system is critical for patients after RC. Therefore, prior investigations attempted to apply standardized perioperative care including Enhanced Recovery after Surgery (ERAS) protocols for patients treated with RC that resulted in some reduction in length of hospitalization^11–13^. A couple of recent studies regarding the effect of gum chewing showed a significant decrease in length of hospitalization by promoting prokinetics of GI system and its functional recovery^14,15^. A review study of prokinetics pharmacologic medications in patients with PI showed that some prokinetics significantly decreased the time to flatus and length of hospitalization^16^. Moreover, patient controlled intravenous analgesia (PCIA) is the most common methods of opioid administration for the management of postoperative pain after major surgeries^17^, but adverse effects of opioids such as drowsiness, respiratory depression and GI dysfunction continuously increased the requests of alternative analgesics for PCIA^18^. Nonsteroidal anti-inflammatory drugs (NSAIDs) have been a reasonable alternative analgesic for opioids and it reduced postoperative morphine consumption by 30–50% in many clinical trials that consecutively lead to a decrease in morphine adverse effects^19^. Ketorolac tromethamine is one of the clinically available intravenous NSAIDs with a better analgesic efficacy that might replace opioids for PCIA on postoperative pain management. In addition, ketorolac shortened the duration of bowel immobility after major bowel surgery when it was added to PCIA^20^.

Thus, this study investigated the effect of Ketorolac as the main analgesic of postoperative PCIA in patients undergoing RC due to bladder cancer, with a hypothesis that ketorolac might decrease adverse effects of morphine PCIA.

## Methods

### Patient selection and study design

The present study was a single center prospectively designed comparative trial of eligible patients who underwent RC with pelvic lymphadenectomy for the treatment of high grade NMIBC or MIBC from November 2003 to May 2017. An approval of this study was obtained from Seoul National University Bundang Hospital Institutional Review Board (B-1710/427-101), and written informed consent was acquired from all patients before the inclusion to the research. Moreover, all research was performed in accordance with relevant research regulations. Among the RC patients, the patients with history of previous abdominal surgery, inflammatory bowel disease, neoadjuvant chemotherapy or radiotherapy, known allergy or contraindication to opioid and NSAIDs, chronic kidney disease stage ≥2 with glomerular filtration rate (GFR) <90 ml/min/m^2^ and history of bleeding disorder were excluded during the research design. The patients were randomly allocated to one of the two groups according to the types of PCIA that would be administered. The randomization ratio between two study groups was 1:1, where one group received ketorolac based PCIA (NSAIDS group) with initial dose of 60 mg bolus and 5 mg/h continuous basal intravenous (IV) infusion and the other group received morphine based PCIA (morphine group) with initial dose of 5 mg bolus and 2 mg/h continuous basal IV infusion. In addition, patients were instructed before RC to use PCIA if the postoperative pain scale was ≥4 in numerical rating score (NRS).

### Surgical procedures and perioperative management

RC was performed by 3 experienced surgeons who executed more than 30 cases RC before the recruitment to the study. The surgical modalities performed for bladder cancer treatment were open radical cystectomy (ORC) with ileal conduit (IC), ORC with orthotopic neobladder (ON), robot assisted radical cystectomy (RARC) with IC, and RARC with ON. The surgical methods were decided by the patient’s preference and a comprehensive discussion regarding the surgical benefits, complications and alternative operative methods was undertaken between the patient and attending surgeon. Two days before RC, patients had a clear liquid diet and the day before RC, patients underwent fasting and bowel preparation with non-intestinal absorbing antibiotic solutions. In ORC, a 12–14 cm midline vertical incision was made and RARC accompanied 7 cm size midline incision for extracorporeal urinary diversion. In cases of RARC with ON, bowel harvesting and neoveicourethral anastomoses were performed by open technique with 7 cm midline incision to minimize operative time. Pelvic lymph node dissection was performed in every patient from the level of obturator fossa, and internal/external/common iliac lymph nodes up to the ureteroiliac junction. In addition, the dorsolateral peritoneal layer was meticulously repaired to promote early bowel function recovery. All RC patients received 1~2 µg/kg of IV sufentanyl immediately after the surgery while they are in postoperative recovery room. Perioperative antibiotic treatment included aminoglycoside, 3^rd^ generation cephalosporin, and metronidazole for 48 hours after RC and 3^rd^ generation cephalosporin single therapy was marinated until discharge from the hospital. Anti-emetics were not routinely administered and no other prokinetic medications were given to the patients after surgery.

No patient received any special nutrition solution before RC and total parenteral nutrition (TPN) consisted of Nutriflex lipid peri (B. Braun Medical, Melsungen, Germany) containing glucose, lipids and amino acids with total energy of 955 kcal/1250 ml was administered to all patients since the postoperative day 1 until patients started to have a soft diet. Additional glucose 5% IV solution was administered for the first 4–5 days after RC in both study groups. Nasogastric tube was removed immediately after RC and clear liquid diet started after the recovery of active bowel sound was confirmed by auscultation. Soft diet resumed after the full functional recovery of GI system, which was confirmed by the return of the first flatus. For uncontrolled severe breakthrough pain management, additional intravenous paracetamol 2 g was given. Patients were discharged when they are in stable condition with no specific complications observed.

### Operative outcomes and statistical analysis

Basic characteristics of the patients including proportions of gender, age, presence of DM, BMI, Charlson comorbidity index, pathologic stage, preoperative estimated GFR (eGFR), operation time, intraoperative blood loss, and surgical types were evaluated and compared between ketorolac and morphine group. The study endpoint was time to recovery of bowel function, which was defined by the first passage of flatus and bowel movements after RC. The short term postoperative complications, which occurred during the first 30 days after RC, were represented by the modified Clavien classification^21^. Early complications were also analyzed according to complication subtypes. GI complications including postoperative PI requiring nasogastric tube replacement and severe colitis requiring TPN administration were also evaluated. Moreover, pain intensity for the first 48 hours after RC was depicted in NRS, and evaluated in both groups. According to the initial analysis of preliminary data, 70 patients were needed for a statistical evaluation to detect variances between two PCIA groups in terms of time to first bowel movement and flatus with 95% power and a two sided probability of 0.05. All statistical analyses were performed with SPSS version 22.0 (SPSS, Chicago, IL), and p = 0.05 was considered statistically significant. The Student t-test, Mann–Whitney U test or the Pearson chi-square test were used to compare the basic characteristics and the postoperative outcomes of the two study groups. The time to linear mixed models for repeated measures was performed to analyze the mean changes of pain intensity presented in NRS during the postoperative 48 hours.

## Results

**Figure 1:**
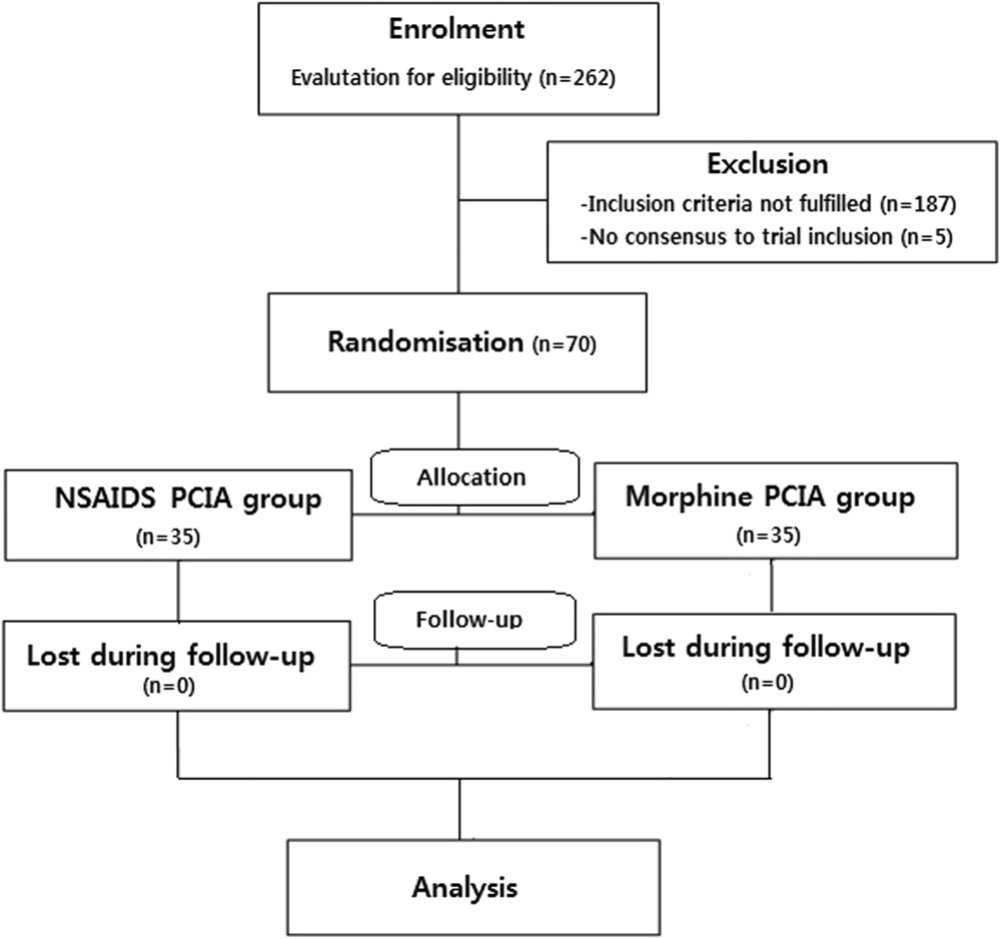
Consort diagram showing the numbers of patients who were randomly assigned to each study groups.

Figure 1 shows a consort diagram describing the number of patients who were randomly assigned to the study. A total of 262 consecutive patients were reviewed and 192 patients were excluded because 187 patients did not meet inclusion criteria and 5 patients refused to enrolment of the study. 70 patients fulfilled the inclusion criteria and 35 patients were assigned to the NSAIDS group and the other 35 patients were assigned to the morphine group. The randomized allocation of the cohorts was conducted by one of the investigators involved in this study and this simple randomization was performed under no limitations by applying NSAIDS PCIA with a consideration of the cohort size in each study group. Overall, all of the corresponding 70 patients completed the study.

Preoperative patient characteristic are shown in Table 1 and there were no significant differences observed in the corresponding variables between the NSAIDS and the morphine group. Nevertheless, the morphine group had 7 patients who received preoperative anticoagulation by oral aspirin, whereas 3 patients of the NSAIDS group had oral aspirin for preoperative anticoagulation (p = 0.006). All of the patients who received preoperative anticoagulation therapy stopped taking aspirin 1 week before the surgery and there was no patient received other form of anticoagulation therapy within the study cohort. A relatively even distribution of urinary diversion types was seen and both groups had the greatest proportions of patients underwent RARC with IC among the four surgical methods (NSAIDS group 48.6%, morphine group 51.4%, Table 1). The mean time to flatus and the first bowel movement were significantly decreased in the NSAIDS group compared to the morphine group (mean time to flatus: p = 0.028/NSAIDS group 2.3 ± 0.9 days/morphine group: 2.7 ± 0.4 days, time to first bowel movement: p = 0.001/NSAIDS group 3.1 ± 0.6 days/morphine group 3.6 ± 0.5 days). The mean time to first clear liquid diet in the morphine group was 3.9 ± 0.6 days, which was significantly greater than 3.5 ± 0.5 days of the NSAIDS group (p = 0.002). However, there was no significant difference regarding length of hospitalization between the NSAIDS and the morphine group (15.7 days vs. 16.5 days, p = 0.427).

**Table 1:** Demographics and surgical outcomes of the NSAIDS group and the morphine group.

	NSAIDS group (n = 35)	Morphine group (n = 35)	*p* value
**Demographics and surgical outcomes of the patients**			
Sex			0.460
Men, n (%)	31 (88.6)	32 (91.4)	
Women, n (%)	4 (11.4)	3 (8.6)	
Age (years), mean ± SD (range)	69.4 ± 10.8 (40–86)	69.3 ± 11.8 (40–87)	0.983
DM, n (%)	5 (14.3%)	4 (11.4%)	0.482
BMI (kg/m^2^), mean ± SD	23.7 ± 3.0	22.9 ± 2.8	0.745
Pathologic stage			0.711
CIS, TO, T1	7 (20)	9 (25.7)	
T2	16 (45.7)	14 (40)	
≥T3	12 (34.3)	12 (34.3)	
Preoperative eGFR (ml/min/m^2^), mean ± SD	97.1 ± 6.6	98.5 ± 7.2	0.574
Charlson cormorbidity index			0.643
0–2, n (%)	32 (91.4)	33 (94.3)	
>2, n (%)	3 (8.6)	2 (5.7)	
Pre-operative anticoagulation by oral aspirin	3 (8.6)	7 (20)	0.006
Surgical methods			0.060
ORC with IC	3 (8.6)	7 (20)	
ORC with ON	4 (11.4)	5 (14.3)	
RARC with IC	17 (48.6)	18 (51.4)	
RARC with ON	11 (31.4)	9 (25.7)	
Operational time (min.), mean ± SD	406.3 ± 141.1	409.9 ± 133.1	0.914
Intraoperative blood loss (ml), mean ± SD	497.1 ± 493.5	510.1. ± 472.8	0.784
**Postoperative outcomes of radical cystectomy**			
Postoperative vomiting, n (%)	3 (8.6)	15 (42.9)	**0.001**
Postoperative blood transfusion, n (%)	6 (17.1)	5 (14.3)	0.743
Postoperative eGFR (ml/min/m^2^), mean ± SD	94.8 ± 4.9	96.7 ± 5.3	0.501
Post-Preoperative eGFR (ml/min/m^2^), mean ± SD	2.4 ± 1.6	2.0 ± 1.2	0.476
Time to flatus (days), mean ± SD	2.3 ± 0.9	2.7 ± 0.4	**0.028**
Time to the first bowel movement (days), mean ± SD	3.1 ± 0.6	3.6 ± 0.5	**0.001**
Time to the first clear liquid diet (days), mean ± SD	3.5 ± 0.5	3.9 ± 0.6	**0.002**
Length of hospitalization (days), mean (range)	15.7 (11–28)	16.5 (11–29)	0.427
Complications by modified Clavien classificaiton, n (%)	7 (20.0)	11 (31.4)	0.588
Grade I	1 (2.9)	2 (5.7)	
Grade II	6 (17.1)	8 (22.9)	
Grade III	0 (0.0)	1 (2.9)	
GI complication rate (%)	2 (5.7)	4 (11.4)	0.050
No. of patients satisfied to pain management, n (%)	17 (48.6)	19 (54.3)	0.632

ORC: open radical cystectomy, RARC: robot assisted radical cystectomy, IC: ileal conduit, ON: orthotopic neobladder, eGFR: estimated glomerular filtration rate, GI: gastrointestinal Postoperative eGFR was measured on postoperative day 5. Oral aspirin for anticoagulation was stopped at least 1 week before surgery.

6 and 7 patients received postoperative blood transfusion in the NSAIDS and morphine groups, respectively (p = 0.743). In terms of postoperative renal function, both study groups showed slight decreases in eGFR compared to their preoperative values, but no significant difference were observed between two groups (post-preoperative eGFR: NSAIDS group 2.4 ± 1.6 ml/min/m^2^, morphine group 2.0 ± 1.2 ml/min/m^2^, p = 0.476). In addition, there were 7 patients (20.0%) with postoperative complications in the ketorolac group and the morphine group had 11 patients (31.4%) with postoperative complications.

Clavien classification of surgical complication is shown in Table 1 and the lists of disease entities observed after RC are presented in Table 2. There were 2 cases of urethral anastomosis leakage in grade I complications, which were managed by increasing indwelling period of urethral catheter up to 3 weeks or greater. There were 5 cases of UTI, 3 cases of uncontrolled nausea requiring anti-emetics, 3 cases of PI, and 2 cases of sepsis in grade II complications, which were solely managed by medical therapy. Grade III complications (postoperative hematoma and consecutive wound dehiscence) were observed in only one patient of the morphine group. The patient with grade III complications underwent surgical treatment and fully recovered afterwards. Although there was no significant difference between the two study groups in terms of overall GI complications (p = 0.050, Table 1), the morphine group had greater proportions of patients with PI and severe nausea (Table 2). The patients with severe nausea in both study groups were managed with a single use of antiemetic drug (0.3 mg of intravenous ramosteron). The patient with PI in both NSAIDS and morphine groups received nasogastric tube insertion and the complication resolved spontaneously within three days after the tube insertion.

**Table 2:** 1 patient in the morphine group had both postoperative hematoma and wound dehiscence.

Complication, n(%)	NSAIDS group (n = 7)	Morphine group (n = 11)
**Complication types after radical cystectomy**		
Urinary tract infection	2 (28.6)	3 (27.3)
Severe nausea	1 (14.3)	2 (18.2)
Ileus	1 (14.3)	2 (18.2)
Urinary leakage	1 (14.3)	1 (9.1)
GI bleeding	0 (0.0)	0 (0.0)
Sepsis	2 (28.6)	2 (18.2)
**Surgical treatment required complications**		
Small bowel obstruction	0 (0.0)	0 (0.0)
Postoperative hematoma	0 (0.0)	1 (9.1)
Wound dehiscence	0 (0.0)	1 (9.1)

**Figure 2:**
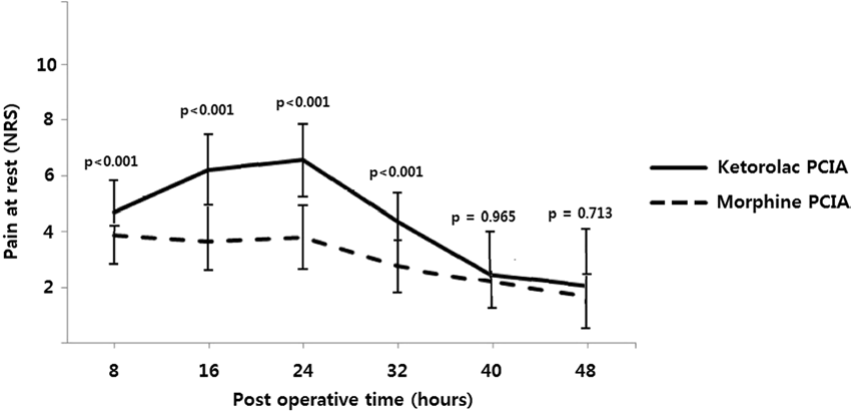
Numerical rating scores (NRS) for postoperative pain evaluation after radical cystectomy. Values are shown as means and 95% confidence interval (represented by the lines extending above and below each plot).

Regarding the postoperative pain management, the morphine group showed a slightly greater number of patients satisfied with analgesic effect of PCIA compared to the ketorolac group, but it was statistically insignificant (morphine group: 54.3%, ketorolac group: 48.6%, p = 0.632). Postoperative NRS pain assessments for 48 hours after RC showed that the use of ketorolac or morphine for PCIA was associated with tolerable reductions in pain scores. Moreover, NRSs showed significantly better analgesic efficacy in the morphine groups until 32 hours after surgery, but the pain scale of both study groups became similar after 40 hours relapsed from RC (Fig. 2).

The differences in the variables related to bowel function recovery, which are stratified according to the surgical modalities, are shown in Table 3. No matter with the types of surgery (ORC or RARC) underwent, the ketorolac group showed a significant reduction in postoperative vomiting rate, the mean time to flatus, the mean time to first bowel movement, and the mean time to first clear liquid diet compared with the morphine group. Both ORC and RARC cases showed no significant difference between the ketorolac and the morphine group in terms of GI complication rate (ORC: p = 0.051, RARC: p = 0.304, Table 3).

**Table 3:** NSAIDS: nonsteroidal anti‐inflammatory drugs, GI: gastrointestinal, SD: standard deviation.

Comparison of bowl function recovery betwen different surgical methods			
Open radical cystectomy	NSAIDS group (n = 7)	Morphine group (n = 8)	*p* value
Postoperative vomiting, n (%)	2 (28.6)	8 (100)	**0.026**
GI complication rate (%)	2 (28.6)	3 (37.5)	**0.051**
Time to flatus (days), mean ± SD	3.6 ± 0.2	4.2 ± 0.8	**0.001**
Time to the first bowel movement (days), mean ± SD	4.1 ± 0.2	4.6 ± 0.2	**0.022**
Time to the first clear liquid diet (days), mean ± SD	4.0 ± 0.9	4.5 ± 0.8	**0.001**
**Robot assisted radical cystectomy**	**NSAIDS group (n = 28)**	**Morphine group (n = 27)**	***p*** **value**
Postoperative vomiting, n (%)	1 (3.6)	7 (25.9)	**0.003**
GI complication rate (%)	0 (0.0)	1 (3.7)	0.304
Time to flatus (days), mean ± SD	1.9 ± 0.7	2.6 ± 0.3	**0.001**
Time to the first bowel movement (days), mean ± SD	2.9 ± 0.5	3.4 ± 0.4	**0.001**
Time to the first clear liquid diet (days), mean ± SD	3.4 ± 0.4	3.7± 0.3	**0.001**

## Discussion

RC with pelvic lymph node dissection and urinary diversion is an extensive surgical procedure that results in a significant rate of postsurgical complications^7^. RC is different from most bowel surgery as RC deperitonealizes the pelvic cavity for a significant duration to undertake adequate lymph node dissection. Moreover the average age of the patients undergoing RC is older than other major abdominal surgeries, which means a higher rate of comorbidity can be accompanied^22,23^. Some studies suggested that the presence of urine in the surgical field might hinder the recovery of colonic motility^24^. In addition, significant increases in sympathetic activity and concentration of plasma catecholamine in postsurgical state might be also promoting factors of PI^25^. Postoperative PI is generally defined as the absence of intestinal movements or contractions for a transient period, but the definition is observer dependent and it varies substantially in many cases. Some researches even use other confusing nomenclature such as subileus^26^. According to the demographic study of Goldstein *et al*., almost $1.42 billion costs annually in the United States for treating postoperative PI^27^ and reducing the duration of postoperative PI is a critical factor to minimize the medical costs for patients undergoing RC. In the present study, the incidence of postoperative PI, which required a prolonged fasting and nasogastric tube insertion (≥7 days), was 2.9% in the ketorolac group and 5.7% in the morphine group. This result is lower than data reported by other prior studies (12–25.4%)^11,28,29^, and we speculate that a reduced PI rate in this study is because of a different definition of postoperative PI applied to the study, as PI does not have a standard definition.

In general, since there is a scanty evidence of bowel preparation reducing the frequency of postsurgical complications, a recent trend is for no preparation if only small bowel was used^30^. However, we performed conventional types of mechanical bowel preparation in all RC patients on the day before surgery due to high consumption of vegetables in Korean population where dietary fibers in vegetables might increase in residual volume of stool. Routine use of nasogastric tubes in postsurgical state is not required after RC because nasogastric tube insertion does not prevent postoperative PI^31^. We removed nasogastric tube after surgical procedures finished, but reinserted the tube only when there was postoperative PI diagnosed. Early restoration of oral feeding has been recommended in many previous researches^31,32^, yet possible risk of intestinal anastomotic leakage, which occurs up to 20% of all colorectal surgery cases, prevents early feeding to be accepted in all RC patients^33^. In the present study, liquid diet was not given to the patients until active bowel movement was restored. Our results showed that the ketorolac group had significantly reduced time to the first liquid diet compared to the morphine group. Compared to the morphine group, the ketorolac group showed better outcomes in all of the variables related to bowel function recovery including the time to flatus and the time to the first bowel movement. We believe this result was due to intestinal hypokinesis caused by opioid PCIA use in the morphine group. Adverse effects of morphine might be regarded as a limitation of morphine PCIA. Walder and associates^18^ showed 31% of the patients receiving opioid-based PCIA experienced severe GI troubles such as nausea and vomiting that made the patients to discontinue using opioid-based PCIA.

Previous researches showed that many NSAIDs increase the risk of perioperative bleeding^34^. An increase in bleeding diathesis is prominent with non-selective COX inhibitors including ketorolac because they interfere with platelet aggregation^35^. However, our study results were not consistent with these findings as the incidence of postoperative blood transfusion showed no difference between the ketorolac and the morphine group. There was a patient who had hematoma formed on incision site in after ORC that caused wound dehiscence requiring surgical intervention, but it should be recognized as the result derived from a complication of open abdominal surgery rather than ketorolac caused bleeding diathesis. Thus, we believe that ketorolac does not increase postoperative bleeding risk after RC, but it still needs to be further discussed in future researches.

Renal toxicity is one of the most common adverse effects of NSAIDS as it can result in reversible acute kidney injury caused by vasoconstriction, and GFR might be decreased within days after the start of NSAIDS^36^. Our study results showed that both NSAIDS and morphine groups had only a slight decrease in eGFR measured on postoperative day 5 compared with their preoperative eGFR. Although the NSAIDS group showed a greater decrease in postoperative eGFR than the morphine group, the difference between two study groups was statistically insignificant. In addition, the whole study cohorts were given multiple medications including triple antibiotics therapy during the first 48 hours after the surgery. Thus, we believe that NSAIDS may not be the sole and significant contributor of decrease in eGFR after RC in this study.

There are few studies reported that NSIADs are associated with a reduction in length of hospitalization^37^, but our study results showed no significant difference between two study groups. Since there are multiple factors related to hospitalization length including the medical costs of patients, a relatively faster bowel recovery effect of ketorolac PCIA might be compensated by the influences of other perioperative factors in the present study. Especially, many patients in Korea ask doctors to postpone discharge even after their full recovery because the national healthcare system covers most of the medical costs, which enables the patients to pay only 5 to 10% of total medical costs. Thus, the request of patients might have influenced hospitalization length substantially in the present study. Moreover, the effect of prokinetic agents to bowel function recovery after RC needs to be studied in further studies as no prokinetic medications used in this study.

Although there has been a tremendous progress in analgesia techniques and understanding pathophysiology of pain, postoperative pain still remains one of the most bothersome problems of major abdominal surgery. In addition, prior researches showed that insufficient pain management is associated with negative postsurgical outcomes including prolonged GI dysfunction and respiratory complications^38^. Our study results showed that both ketorolac and morphine were effective in postoperative pain management during the first 48 hours after RC. Approximately 32 hours after RC, ketorolac PCIA had similar analgesic efficacy compared with morphine PCIA according to NRS of postoperative pain analysis. Prior studies showed that RARC was associated with a reduction in the time to flatus and bowel movement compared with ORC (flatus: 2.3 days vs. 32. days/p < 0.001, bowel movement: 3.2 days vs. 4.3 days/p = 0.008)^39^. Our results showed that the variables related to postoperative bowel function recovery were similar regardless of the operational modalities.

The limitation of our study includes the exclusion of other active comparable NSAIDS or non-opioid analgesics. Future studies need to compare NSAIDs, including selective COX-2 inhibitors, with different types of opioid analgesics. Morphine sparing effects of ketorolac in combination with PCIA should be also discussed in further research. Moreover, relatively small study cohort can be a limitation of this study. Despite the limitation, the present study results suggest significant benefits of ketorolac PCIA in terms of promoting postoperative bowel recovery after RC.

## Conclusion

According to the results of our study, ketorolac as PCIA is relatively safe and effective in pain management. It also provided better bowel function recovery after RC regardless of surgical modalities. However, there was no difference in length of hospitalization between the ketorolac and the morphine group at least with Korean population.
